# Optimization of the Filament Winding Process for Glass Fiber-Reinforced PPS and PP Composites Using Box–Behnken Design

**DOI:** 10.3390/polym16243488

**Published:** 2024-12-14

**Authors:** Sevinc Orman, Mustafa Dogu, Belma Ozbek

**Affiliations:** 1 Department of Chemical Engineering, Yildiz Technical University, Davutpasa Campus, Esenler 34220, Türkiye; ormansevinc@gmail.com; 2 Mir Arastirma ve Gelistirme Inc., Esenyurt 34517, Türkiye; mus.dogu@gmail.com

**Keywords:** filament winding, thermoplastic composites, glass fiber, experimental design, process optimization

## Abstract

Filament winding is a widely used out-of-autoclave manufacturing technique for producing continuous fiber-reinforced thermoplastic composites. This study focuses on optimizing key filament winding process parameters, including heater temperature, roller pressure, and winding speed, to produce thermoplastic composites. Using Box–Behnken response surface methodology (RSM), the study investigates the effects of these parameters on the compressive load of glass fiber-reinforced polypropylene (GF/PP) and polyphenylene sulfide (GF/PPS) composite cylinders. Mathematical models were developed to quantify the impact of each parameter and optimal processing conditions were identified across a wide temperature range, enhancing both manufacturing efficiency and the overall quality of the composites. This study demonstrates the potential of thermoplastic filament winding as a cost-effective and time-efficient alternative to conventional methods, addressing the growing demand for lightweight, high-performance, out-of-autoclave composites in industries such as aerospace, automotive, and energy. The optimized process significantly improved the performance and reliability of filament winding for various thermoplastic applications, offering potential benefits for industrial, aerospace, and other advanced sectors. The results indicate that GF/PPS composites achieved a compressive load of 3356.99 N, whereas GF/PP composites reached 2946.04 N under optimized conditions. It was also revealed that operating at elevated temperatures and reduced pressure levels enhances the quality of GF/PPS composites, while for GF/PP composites, maintaining lower temperature and pressure values is crucial for maximizing strength.

## 1. Introduction

Filament winding is an efficient, single-step, in situ production technique for continuous fiber-reinforced polymer composites. It enables the manufacturing of complex-shaped (axisymmetric and non-axisymmetric) and closed-form structures in a relatively short time. It can be applied to both thermoset and thermoplastic composites, although most applications involve thermosets [1,2].

Thermoplastics, however, stand out due to their high strength, toughness, chemical resistance, unlimited shelf life, and recyclability. Unlike thermosets, thermoplastics can be reshaped with varying temperatures, and they do not require a curing step, which means faster processing and eligibility for automation. Generally, the use of thermoplastics and their composites is limited due to their processing difficulties arising from their high viscosity. Thermoplastics have a higher viscosity than thermosets, even in the molten state [3,4,5,6,7]. Thermoplastic applications have been popular in recent years due to the evolving machinery and control software technologies. It is now possible to manufacture filament-wound thermoplastic composites in a very short time with robotic arms implemented with laser heaters [8,9,10,11,12]. In the filament winding process of thermoplastics, generally, prepreg tapes and hybrid yarns are used to ensure the impregnation of matrix polymer to the reinforcing fibers [4,5,6,13,14,15]. The compaction roller helps prevent the formation of entrapped air bubbles and voids between the layers subjected to a heat source. As thermoplastics do not require a curing stage, production cost, time, and energy consumption are significantly reduced [1,2,4,16].

Although there are several studies available on thermoplastic fiber placement technology, research on the thermoplastic filament winding process is quite limited. Radford et al. [6] studied the applicability of a fused deposition hot-end system combined with the filament winding method. Duan et al. [17] studied the filament winding modeling of a carbon fiber-reinforced pressure vessel with an HDPE liner by using an ANSYS ACP module. A reliable finite element model to be used for further simulations was obtained. Mindermann et al. [18] explored multi-stage coreless filament winding to design hybrid fiber composite/metal structures, achieving tensile strengths up to 800 MPa with a 30% weight reduction compared to traditional metal components. Wong et al. [19] studied the process parameters of aramid-reinforced PA6 composites produced by the filament winding method. Commingled yarns were used in the winding process and the effects of heat gun temperature, line speed, fiber tension, compaction force, and preheater temperature were investigated. Dell’Anna et al. [20] investigated the potential use of E-glass (GF)-reinforced PET prepregs in composite pipe production. The filament winding system was integrated with an ultrasonic heater. It was emphasized that GF/PET prepreg tapes can successfully be used for composite pipe production by ultrasonic filament winding. In the authors’ previous work [21], they studied the ultrasonic filament winding process of commingled thermoplastic roving. Fiber impregnation and ply consolidation were included in a single system. The heat transfer modeling was made by finite element (FE) analysis to map temperature distribution in the composite during consolidation. The findings were validated by temperature measurements during static ultrasonic consolidation. Stefanovska et al. [3] investigated the filament winding process of glass fiber-reinforced PP rovings (Twintex^®^ R PP 60 B 1870, Owens Corning, Toledo, Ohio, USA). A prototype filament winding machine made by Mikrosam AD was used. The effects of consolidation roller temperature, second roller temperature, air gun temperature, consolidation roller pressure, and winding speed were examined. The best consolidation was obtained at 150–200 air gun temperature, 5 bar roller pressure, and 1.5 m/min winding speed. Grouve et al. [8] studied carbon fiber-reinforced polyphenylene sulfide (CF/PPS) tapes at a laser-assisted tape-laying machine provided by AFPT GmbH (Dörth, Germany). The effects of various laser power, incident laser angle, and placement velocity on the interfacial fracture toughness were measured by the mandrel peel tests. The results were compared to press-consolidated specimens, and it was concluded that excellent bond quality can be obtained at high velocities and low input power.

Recent studies have highlighted advancements in filament winding for thermoplastic composites, focusing on process optimization and mechanical performance. Karbuz et al. [22] explored the production of hexagonal cross-section thermoplastic composite parts using glass fiber-reinforced polypropylene (GF/PP) prepreg tapes and an automated filament winding system. Through the Taguchi experimental design, the critical parameters—heater temperature, compression roller pressure, mandrel rotation speed, and corner radii—were optimized to achieve maximum interlaminar shear strength (211 MPa) and compressive strength (49.40 MPa). Their findings highlight that corner radius significantly impacts the mechanical performance of non-cylindrical geometries, offering insights into the limitations and opportunities in adapting filament winding for complex shapes. Similarly, Deng et al. [23] investigated the influence mechanisms and optimization of prepreg tape winding parameters. Using carbon fiber-reinforced polyamide 66 (CF/PA66) tapes, they employed response surface methodology to identify optimal combinations of heating temperature, tape tension, and roller pressure. This resulted in maximized tensile strength and highlighted the critical impact of these parameters on mechanical performance. Geng et al. [24] investigated the effects of laser-assisted winding on CF/PPS composites, achieving a maximum tensile strength of 2571.51 MPa by optimizing the core mold temperature, winding rate, and laser power. Vedernikov et al. [25] demonstrated that composites made from commingled yarns exhibited 106% higher flexural strength and 92% higher interlaminar shear strength compared to those made with commercial prepreg tapes. Xian et al. [26] improved the bending properties of GFRPP bars using fixture optimization, achieving a 43.2% increase in bending strength (up to 840 MPa). Chen et al. [27] investigated the process parameter optimization of continuous carbon fiber-reinforced polycarbonate (CCFRPC) prepreg filaments using a melt impregnation method. Their study identified the optimal process parameters as an outlet mold temperature of 285 °C, an impregnation mold temperature of 305 °C, and a winding speed of 1 m/min. These conditions resulted in a tensile strength of 1298 MPa and minimal diameter variability, demonstrating the suitability of their methodology for 3D printing applications.

Compression testing has been highlighted as a critical tool in evaluating and optimizing filament-wound composites, particularly for identifying delamination risks and improving mechanical properties. Mo et al. [28] highlighted how optimized winding sequences enhance compression strength in coreless filament-wound lattice structures. Lisbôa et al. [29] demonstrated that radial compression testing effectively evaluates delamination risks in filament-wound composite cylinders. Mishra et al. [30] explored the axial and radial compression behavior of hybrid tubular composites made from carbon and glass fibers, impregnated with epoxy resin, developed through a robotized filament winding process.

The present study aimed to optimize the manufacturing process of a hot-air filament winding system by investigating the effects of major process parameters. In the literature, studies focusing on the systematic optimization of thermoplastic filament winding processes are highly limited, particularly for GF/PP and GF/PPS composites, where comprehensive analyses of process parameters and their effects on mechanical properties are lacking. Therefore, this study addresses this gap by employing response surface methodology (RSM) and Box–Behnken design to investigate the effects of key process parameters—heater temperature, roller pressure, and winding speed—on the mechanical properties of GF/PP and GF/PPS composites. Using these advanced statistical tools minimizes the required number of experiments while maintaining accuracy and provides a deeper understanding of parameter interactions [31].

An air-blowing heater was employed as the heat source for the filament winding system due to its low cost, accessibility, and ease of implementation, making it a practical choice for enhancing industrial scalability and cost efficiency. GF/PP and GF/PPS were chosen due to their distinct melting points (173 °C for PP and 282 °C for PPS), enabling a broad investigation of process mechanisms. GF/PP, known for its high strength, toughness, and chemical resistance, is widely used in industrial, automotive, and sporting goods applications due to its low cost and ease of processing. PPS, while more challenging and expensive to process, offers flame retardancy and superior mechanical and chemical properties, making it ideal for aerospace and industrial applications. By examining these materials within the same framework, this study demonstrates the versatility of filament winding across a range of industries. Integrating these aspects, the study addresses a critical gap and offers a novel contribution to both academic research and the industrial applications of thermoplastic filament winding.

In summary, this research provides a robust methodology for optimizing thermoplastic filament winding, contributing to the growing demand for lightweight, high-strength composites in modern industries. It addresses key challenges and applies advanced experimental design techniques to facilitate future innovations in composite manufacturing.

## 2. Materials and Methods

### 2.1. Materials

Glass fiber-reinforced polypropylene (GF/PP) and polyphenylene sulfide (GF/PPS) composite tapes, provided by Celanese (Irving, Texas, USA), were used in this study. The Celstran CFR-TP PP GF70-13 tape comprises 70% E-glass by weight and is a continuous fiber-reinforced (unidirectional) PP composite tape. Similarly, Celstran CFR-TP PPS GF60-01 consists of 60% E-glass by weight and is also a continuous fiber-reinforced (unidirectional) PPS composite tape. To facilitate the filament winding process, the composite tapes were cut into 5 mm wide slit tapes. The properties of these tapes, as outlined in their commercial technical datasheets (TDS), are presented in Table 1.

The thermal properties of the composite cylinders were analyzed using the Perkin Elmer DSC 4000, while the compression tests were conducted on the Zwick Universal Testing Machine (Zwick Z250 Allround, ZwickRoell GmbH & Co. KG, Ulm, Germany).

### 2.2. Thermoplastic Filament Winding System

**Figure 1:**
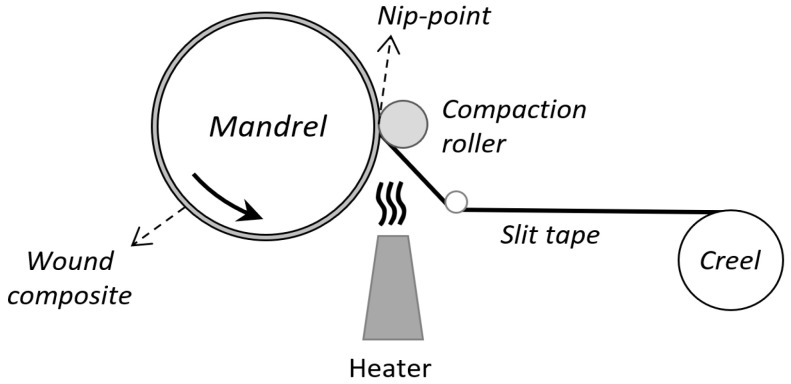
A schematic illustration of the thermoplastic filament winding system used in the present study.

Experimental studies were conducted using a custom-built lab-scale filament winding machine developed by Mir Arastirma ve Gelistirme Inc. This machine is equipped with a cylindrical mandrel, a pneumatically controlled compaction roller (32 mm diameter, 28 mm length), an air-blowing heater, an electronically controlled creel, and winding software (CADWIND^®^ V9). The compaction force of the roller is regulated by a pneumatic valve. The set temperature of the air-blowing heater is adjustable and varies for different experiments. The heater nozzle position is fixed within the tape feeding system. Therefore, a similar heating effect on the nip point was assumed. The tension of the slit tapes was maintained constant in all experiments by the electronic creel. The schematic of the thermoplastic filament winding system is illustrated in Figure 1. This apparatus was validated through multiple trial runs to ensure consistent performance. The optimized process parameters identified are universal and not restricted to this custom-built machine, making the findings applicable to other systems. Furthermore, the same filament winding machine was used by Karbuz et al. [22], and it has demonstrated consistent performance and successful application in producing GF/PP composites with hexagonal geometries.

**Figure 2:**
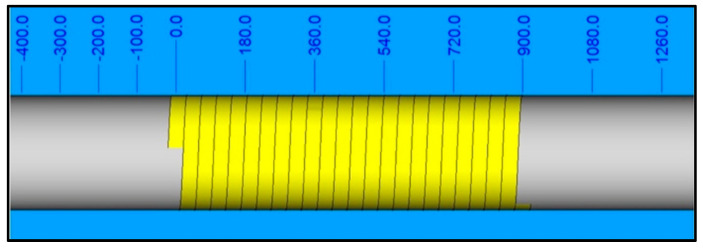
An illustration of the winding pattern created in CADWIND^®^.

Circumferential winding with 100% coverage, as depicted in Figure 2, was carried out according to CADWIND^®^ data. The tension of the slit tape was controlled by the creel and kept constant throughout the process.

**Figure 3:**
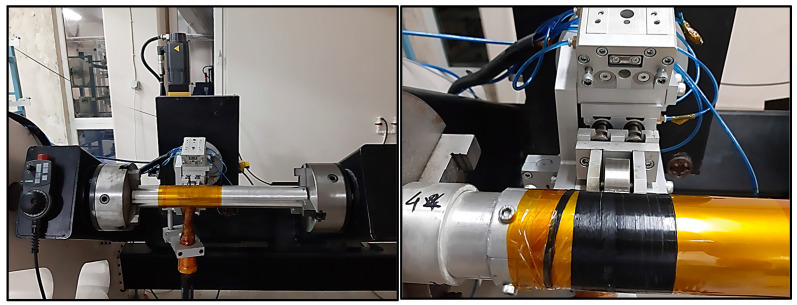
Filament winding system.

**Figure 4:**
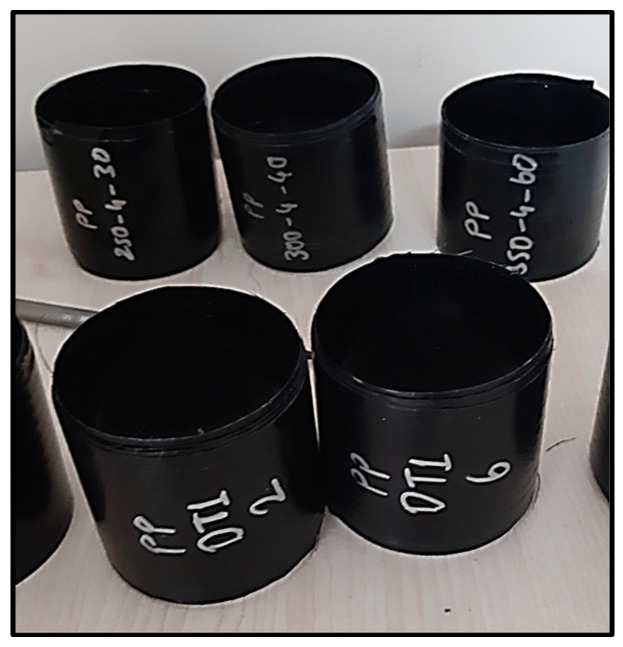
Filament-wound composites.

The filament winding system used in this study and the examples of produced filament-wound cylinders are given in Figure 3 and Figure 4.

The point where the mandrel and compaction roller come into contact is referred to as the nip point. The heat applied creates a polymer melting zone at and around the nip point. The roller exerts pressure on both the incoming tape and substrate, facilitating intimate contact. When intimate contact is achieved at the nip point, the incoming tape and the substrate automatically adhere to each other. To prevent void formation, pressure is applied for a sufficient time, ensuring that the temperature falls below the melting point [32].

### 2.3. Preliminary Studies

It is essential to establish process limits for each material before commencing optimization studies. In preliminary investigations, potential working limits of the processing parameters (set temperature of the heater, compaction roller pressure, and winding speed) were determined based on the adhesion performance between the layers of composite tapes. Three layers of slit tape were consolidated, and attempts were made to separate the layers by hand to evaluate the bond strength. The process limits were selected based on achieving strong adhesion, preventing the layers from being separated manually. Poor adhesion typically occurs when the polymer fails to melt or degrades during consolidation. It was observed that GF/PPS can be processed within a heater temperature range of 550–600 °C, with roller pressures ranging from 4 to 6 bar and winding speeds between 800 and 1600 mm/min. Similarly, GF/PP can be processed within a heater temperature range of 250–300 °C, with roller pressures between 4 and 6 bar and winding speeds ranging from 800 to 1600 mm/min. These processing limits also define the process window, which is a range of parameters that allows for the successful production of the composite. The goal of this study is to determine the optimum point within this process window to achieve maximum composite quality. Moreover, the corresponding linear compaction forces of the pneumatically controlled compaction roller are presented in Table 2.

### 2.4. Methodology

Optimization can be defined as the identification of process conditions that yield the highest possible benefit from a well-defined system within certain constraints [33]. To accurately determine the optimal point of a physical system, it is necessary to examine a large number of independent variables across a wide range [34].

In the filament winding process, the bond strength between each layer is primarily influenced by the heater temperature, roller pressure, and winding speed of the mandrel. Therefore, these three variables were selected as the primary process factors, and response surface methodology (RSM) was utilized to identify the optimal combination that yields the highest mechanical performance, which is measured by compression tests. RSM is a powerful tool that employs statistical and mathematical techniques to characterize a system or process using empirical models derived from experimental design data [33,35,36,37]. These empirical models establish the functional relationship between the dependent and independent variables. Since this relationship is initially unknown, an approximation function is first developed, followed by assessing the data’s compliance with this approach.

**Figure 5:**
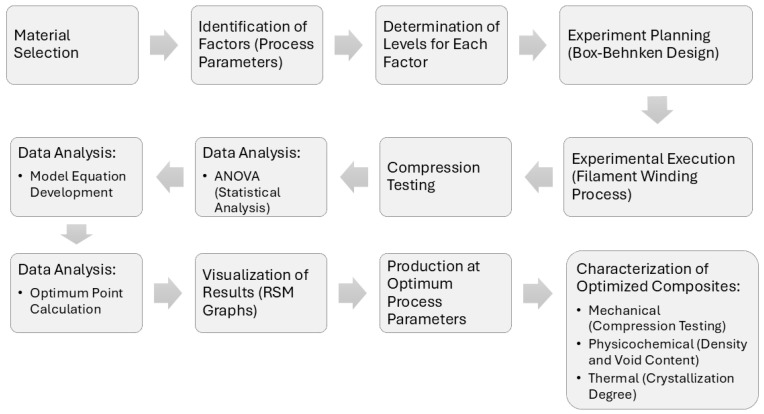
Flowchart of the experimental methodology.

In most RSM problems, first-order or second-order polynomial model approximations are commonly utilized. The least squares method is employed for parameter estimation, and if the fitted surface aligns well with the response function, it indicates that the fitted surface accurately represents the actual system. The proper collection of experimental data is crucial for effective model parameter estimation. Box–Behnken design is a specific experimental design commonly used within RSM. It allows for the efficient exploration of variable interactions while minimizing the number of experiments compared to full factorial designs, making it more cost- and time-effective. Therefore, in this study, the experimentation route was planned and executed based on the Box–Behnken experimental design (3-factor, 3-level, 15 experiments) for both GF/PPS and GF/PP composites [36]. A flowchart that explains the experimental methodology is provided in Figure 5.

Composite cylinders with a 70 mm diameter, 2 mm wall thickness (7 layers), and 55 mm length were manufactured under various process conditions as per the experimental design outlined in Table 3. The process factors and levels of the experimental design are defined in Table 4 and Table 5. The same hoop winding pattern (0°) was employed in all experiments, as depicted in Figure 2.

### 2.5. Compression Tests

The product quality of the filament-wound composites was evaluated through compression tests, which were carried out by the Zwick Universal Test Device (Zwick Z250Allround, ZwickRoell GmbH & Co. KG, Ulm, Germany). Compressive load, a critical indicator of mechanical performance, was selected as the primary response of the experimental design. The bonding strength of the composite layers, reflecting the performance of the filament winding system, was evaluated through compression tests. Compression testing plays a complementary role in optimizing the filament winding process. In many real-world scenarios, filament-wound structures are subjected to radial pressures, such as in pressure vessels or pipelines. By evaluating the bonding quality between layers and identifying potential delamination risks, radial compression tests provide critical insights into the structural integrity of the composites [38,39]. This information enables engineers to fine-tune manufacturing parameters, ensuring that the resulting structures are mechanically robust and suitable for demanding applications.

**Figure 6:**
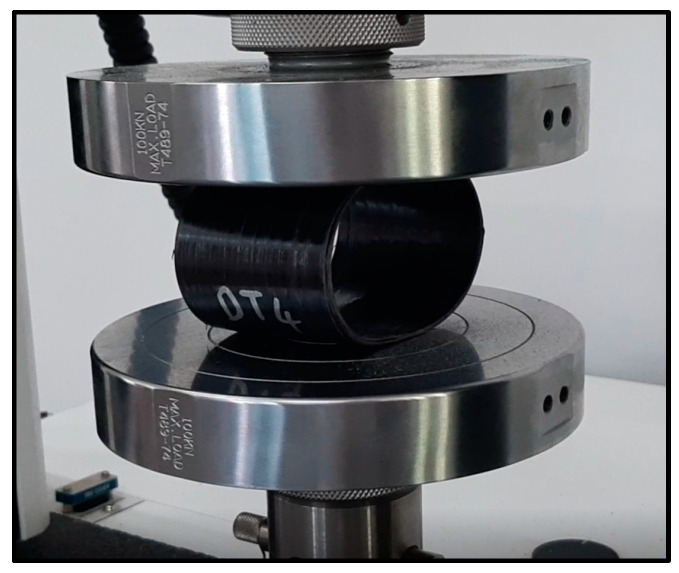
Compression test setup.

By analyzing the compressive load data, the optimal combination of heater temperature, roller pressure, and winding speed was identified to maximize the mechanical performance of the composites. Figure 6 illustrates a compression test applied to composite cylinders.

For each process condition in the experimental design, three cylindrical composites were produced and subjected to compression testing (Figure 6). The test setup was inspired by ASTM D2412-02 [40]. The compression tests were conducted at a speed of 2.5 mm/min until a 3% deflection of the inside diameter occurred. The load corresponding to this deflection was recorded as the compressive load of the composite [39].

A second-order polynomial model approximation and the least squares method were employed to develop a mathematical model elucidating the relationship between the process parameters and the compression test results. The analysis was performed using the trial version of Stat-Ease 360^®^ software, which facilitated the statistical evaluation and optimization of the filament winding process. All necessary calculations, including an ANOVA analysis, coefficient estimation of the model equations, determination of the optimum points, and generation of RSM graphs, were conducted using this software. This comprehensive analysis ensured an accurate and robust optimization of the process parameters, thereby enhancing the reliability and validity of the findings.

### 2.6. Mechanical, Thermal, and Physicochemical Characterization of the Optimized Filament-Wound Composites

In this section, the performance of the filament-wound composites produced at the optimized process conditions is evaluated through mechanical (e.g., compression testing), thermal (e.g., crystallinity analysis via DSC), and physicochemical (e.g., density and void content) characterizations. These tests are essential for verifying the effectiveness of the optimized process parameters and achieving a comprehensive understanding of composite quality.

**Figure 7:**
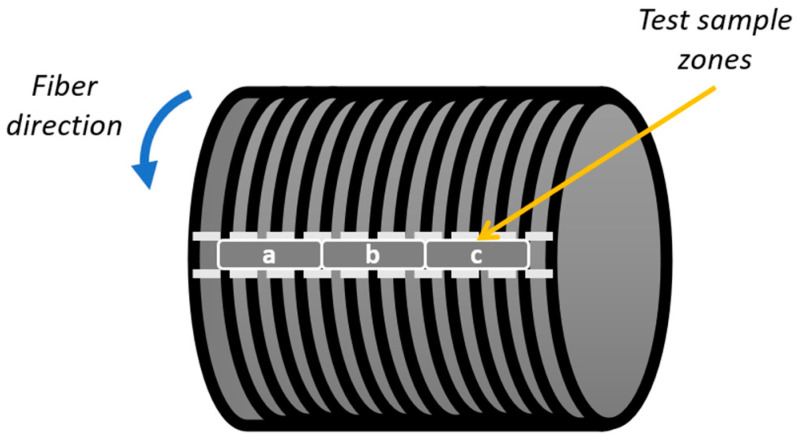
Composite structure and test sampling.

The test samples were collected from three distinct zones (labeled as zones a, b, and c) of the composite cylinders for density measurements and DSC tests, as depicted in Figure 7. For each test, 3 composite cylinders were produced (i.e., GF/PP-1, GF/PP-2, GF/PP-3), and 3 samples were taken from each one of the cylinders (a, b, and c zones in Figure 7).

Archimedes’ principle, as outlined in ASTM D792, was utilized to determine the density of the composites [41]. Given that commercial prepreg tapes were utilized in the composite manufacturing process, the theoretical tape density (ρt) provided in the commercial technical data sheet (TDS) was accepted as the reference density for the analysis (Table 1). According to the commercial TDS, the theoretical density values are 1.885 g/cm3 for GF/PPS and 1.656 g/cm3 for GF/PP.

DSC tests were conducted by using the Perkin Elmer DSC 4000. Heating was performed between 30 °C and 290 °C at a ramp rate of 10 °C/min. The following data were used for the calculation of the crystallinity degree (Xc): the heat of fusion of PPS is 76.5 J/g [42], the heat of fusion of PP is 165 J/g [43], and the resin content of GF/PPS composites is 40%, while GF/PP composites have a resin content of 30%, as indicated in Table 1.

The void content (Vc), which represents the air trapped within the composite structure, can be determined by calculating the difference between the theoretical and measured composite density using Equation (1) [44].

(1) Vc=100−ρc,m (Wrρr+Wfρf)

*W*: weight percentage; *Vc*: void content of composite; *ρ*: density; *c*: composite; *r*: resin; *f*: fiber; *m*: measured; and *t*: theoretical. So, the equation becomes

(2) Vc=100 (1−ρcρt)

Thus, the void content of filament-wound composites was calculated according to Equation (2).

## 3. Results and Discussion

### 3.1. Optimization of Process Parameters

Parallel plate compression tests were conducted on the composites manufactured according to a three-factor, three-level Box–Behnken experimental design, as explained in Section 2.5. The test results, along with the covariance values, are provided in Table 6. The compression test results represent the average of three repeated tests (*n* = 3).

An ANOVA (analysis of variance) was conducted on the compression test results of the composite cylinders to assess the relationship between the process parameters and compressive loads. Fit statistics, indicating the adequacy of the model equations, are reported in Table 7 for both GF/PPS and GF/PP composites. Further results of the ANOVA are presented in Table 8 for GF/PPS and Table 9 for GF/PP composites.

In Table 7, the predicted R^2^ of 0.8913 agrees with the adjusted R^2^ of 0.9802, i.e., the difference is less than 0.2. Adequate precision measures the signal-to-noise ratio. The ratio of 35.894 indicates an adequate signal (a ratio greater than four is desirable) [37].

Table 8 displays the ANOVA results of the GF/PPS experimental design, indicating the statistical significance of the process parameters and their effects on compressive loads. The model F-value of 77.91 implies that the mathematical model is significant. *p*-values less than 0.05 indicate model terms are significant. In this case, A, B, AB, AC, BC, A^2^, B^2^, and C^2^ are significant model terms. Values greater than 0.1 indicate the model terms are not significant. The lack-of-fit F-value of 13.64 implies there is a 6.91% chance that a lack-of-fit F-value this large could occur due to noise and that the lack of fit is not significant. Consequently, this mathematical model can be used to define the design space of the GF/PPS composite production process.

Table 9 illustrates the ANOVA results of the GF/PP experimental design. The model F-value of 267.68 implies the model is significant. There is only a 0.01% chance that an F-value this large could occur due to noise. *p*-values less than 0.05 indicate model terms are significant. In this case, A, B, AB, AC, BC, A^2^, B^2^, and C^2^ are significant model terms. Values greater than 0.1 indicate the model terms are not significant. The lack-of-fit F-value of 1.43 implies the lack of fit is not significant relative to the pure error, meaning the model fits the experimental data. In Table 7, the predicted R^2^ of 0.9759 is in reasonable agreement with the adjusted R^2^ of 0.9942, i.e., the difference is less than 0.2. Adequate precision measures the signal-to-noise ratio. A ratio greater than four is desirable, and the ratio of 51.665 indicates an adequate signal [37]. Therefore, the model can be used to relate process parameters to the compressive load of the GF/PP composites. The coefficient estimation of the quadratic model for the experimental design was carried out based on the ANOVA results. The specific coefficients representing the relationship between the process parameters and compressive loads were calculated, facilitating a deeper understanding of the optimization process for each composite type.

The model equations, which relate the process factors to the compressive load of the GF/PPS and GF/PP composites, are depicted in Equations (3) and (4), respectively. Only the statistically significant factors (*p* < 0.0500) are included in the model equations. These equations are provided in terms of coded factors and can be utilized to predict the compressive load of each composite under specific process conditions (note that only the coded process conditions are applicable in the equations).

R_GF/PPS_ = 3061.51 + 144.00(A) − 83.17(B) + 107.81(AB) + 38.61(AC) − 36.97(BC) − 149.52(A^2^) + 44.64(B^2^) + 130.68(C^2^) (3)

R_GF/PP_ = 2538.09 − 60.81(A) − 85.52(B) − 36.21(AB) − 67.41(AC) − 140.25(BC) + 62.70(A^2^) + 140.52(B^2^) + 41.66(C^2^) (4)

The optimum point within the process window for both GF/PPS and GF/PP composites was determined using the model equations. The calculations were made to reach the maximum compressive load achievable within the process window.

For the GF/PPS production process, the optimum point in terms of coded values is A = 0.09 (≈0), B = −0.99 (≈−1), and C = 1.00. The corresponding actual values are a heater temperature of 575 °C, a roller pressure of 4 bar (equivalent to 321.70 N of compaction force), and a winding speed of 1600 mm/min. The compressive load calculated from the model equation under these conditions is 3356.99 N. These process conditions correspond to the 11th experiment of the Box–Behnken design, resulting in an actual compressive load of 3354.09 ± 4.83% (Table 6). The difference between the calculated and actual value is 2.99 N, which is considered negligible.

**Figure 8:**
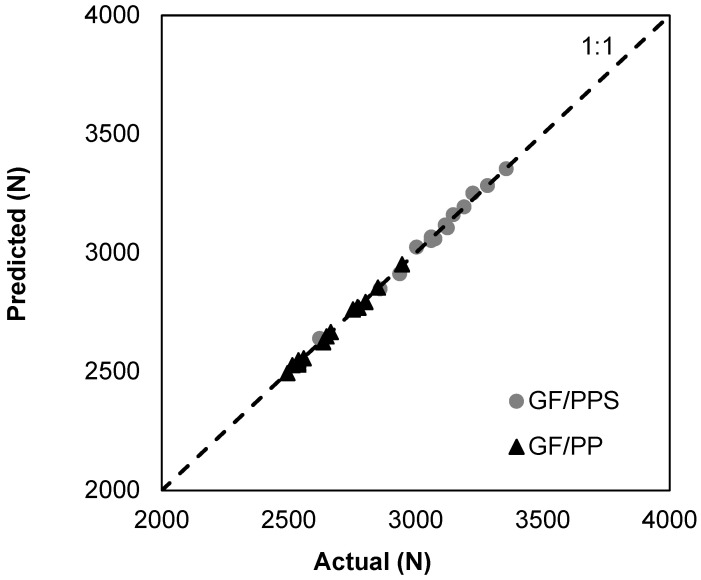
Predicted vs. actual compressive load of GF/PPS and GF/PP composites.

Additionally, the regression coefficients comparing predicted versus actual values are 0.9928 and 0.9973 for GF/PPS and GF/PP, respectively (Figure 8). These coefficients indicate a strong correlation between the predicted and actual values, suggesting the reliability of the model equations in estimating compressive loads.

### 3.2. Effects of Process Parameters on Compression Performance Using Response Surface Graphs

**Figure 9:**
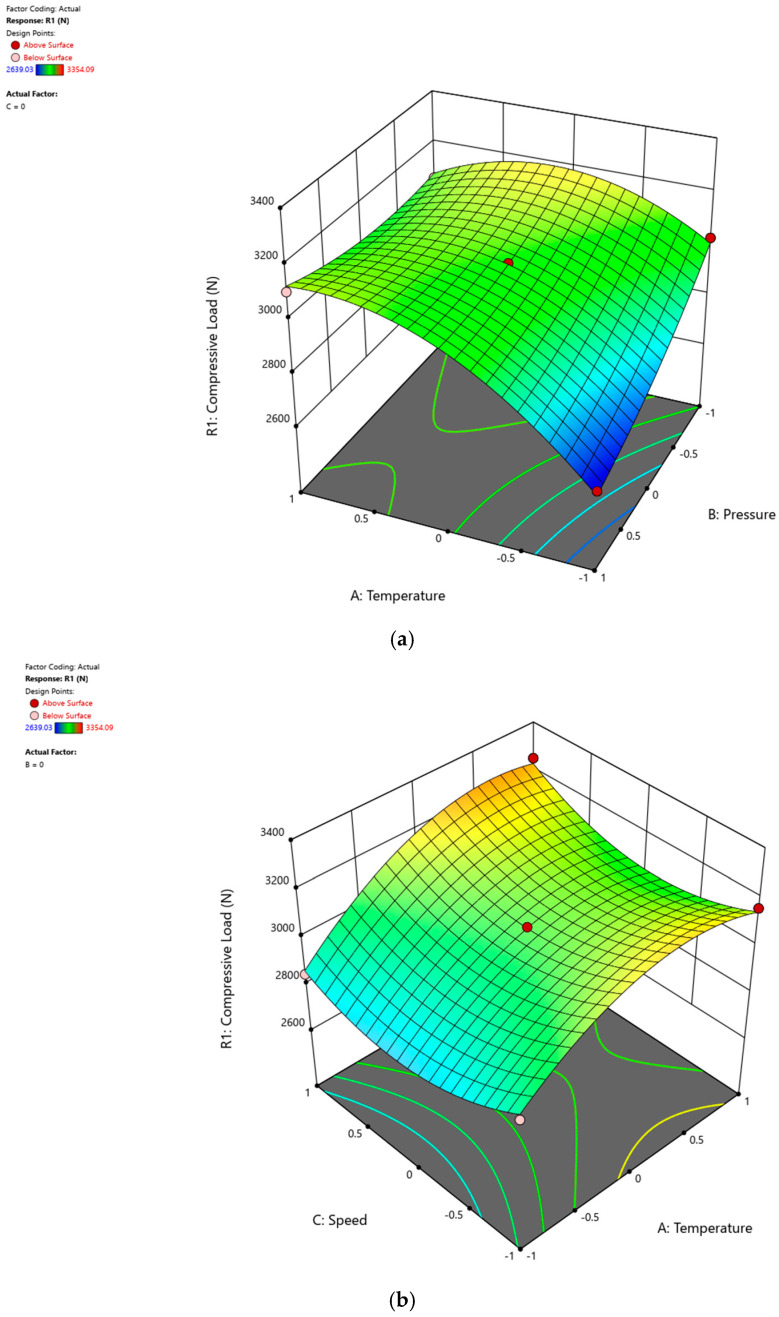

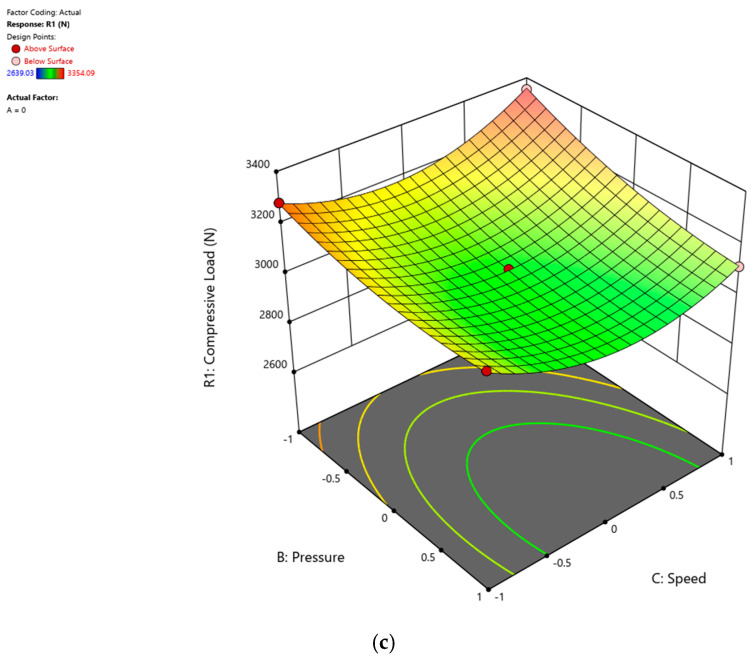
The effects of (**a**) pressure and temperature, (**b**) winding speed and temperature, and (**c**) pressure and winding speed on the compressive load of GF/PPS composites produced by filament winding.

**Figure 10:**
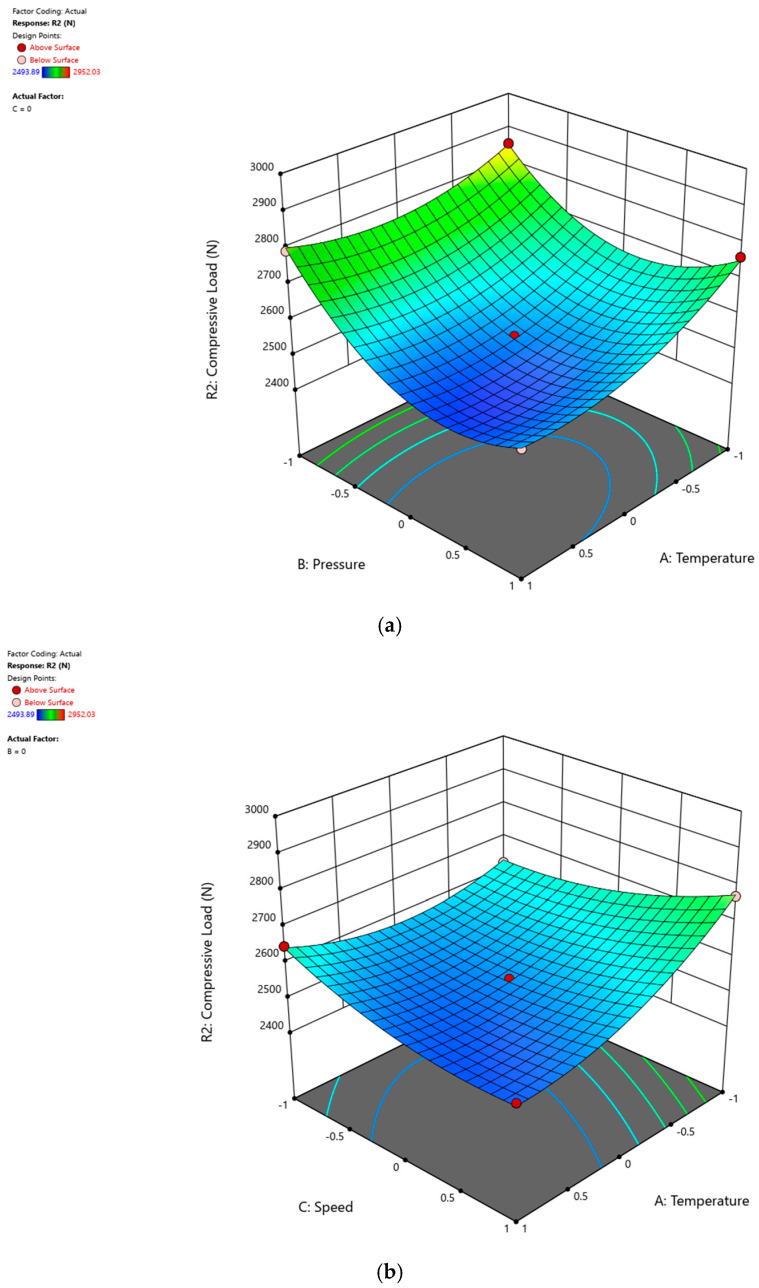

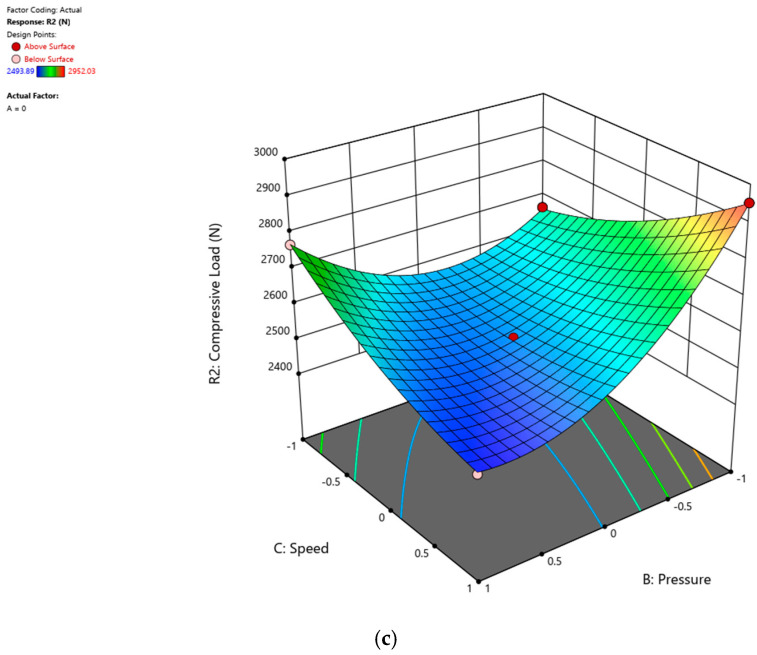
The effects of (**a**) pressure and temperature, (**b**) winding speed and temperature, and (**c**) pressure and winding speed on the compressive load of GF/PP composites produced by filament winding.

The response surface is a three-dimensional graphical representation of the data that facilitates the interpretation and understanding of the relationships between independent and dependent variables. The effects of process parameters on the compressive load of GF/PPS and GF/PP composites are visually represented through response surface graphs in Figure 9a–c and Figure 10a–c. These graphs were generated based on the model equations (Equations (3) and (4)), clearly illustrating how variations in process parameters influence the compressive load. In each graph, the third parameter is constant at the center point, which corresponds to zero, as indicated in Table 4 and Table 5. Keeping one parameter constant enables a more focused analysis of the interactions between the other two parameters and their influence on the response. The central value, representing the midpoint of the experimental range, serves as a balanced reference point, ensuring consistent comparisons across parameter combinations. This systematic approach, combined with the mathematical model equations, comprehensively accounts for the effects of all three parameters, providing detailed insights into their individual and combined impacts on the system response.

The optimal process conditions were determined based on the model equations (Equations (3) and (4)). Figure 9 and Figure 10 are designed solely to visualize the effects of the process parameters on the system’s response. Such visualizations provide valuable insights into the relationships between process variables and the response.

Figure 9a illustrates how roller pressure and heater temperature influence the compressive load of the composites, while winding speed is constant at the center point (zero). It suggests that for high-quality GF/PPS composite production, a higher temperature (A) and lower roller pressure (B) are preferable, with the effect of temperature being more significant than that of pressure. In Figure 9b, roller pressure is constant at the center point (zero) and the effects of winding speed and heater temperature are analyzed. The impact of winding speed (C) is found to be negligible compared to temperature (A), and it is observed that the compressive load of GF/PPS composites increases with rising temperature. Additionally, Figure 9c highlights that lower pressure values are desirable for achieving high compressive loads, with the effect of winding speed (C) being relatively insignificant compared to pressure (B). The heater temperature remains constant (center point) in Figure 9c.

These findings reveal that temperature emerges as the most influential factor, exerting a positive impact on product quality. As the operational temperature approaches the upper limit within the defined process window, there is a notable enhancement in composite strength. Furthermore, roller pressure (or compaction force) emerges as the second most crucial parameter, although with an adverse effect on product quality. Lower roller pressure values contribute to higher composite strength. Given that excessive pressure on fibers can induce deformation, misalignment in fiber orientation, and potential fiber breakage, operating closer to the lower pressure limits of the process window proves to be advantageous. Finally, winding speed demonstrates a minimal influence on product quality compared to temperature and pressure.

In Figure 10a, roller pressure and winding speed change, while heater temperature is kept constant at the center point zero. It suggests that for high-quality GF/PP composite production, a lower temperature (A) and lower roller pressure (B) are preferable, with the effect of roller pressure being more significant than temperature. Figure 10b demonstrates the effects of winding speed and heater temperature on the response, with roller pressure being constant at zero. The impact of winding speed (C) is negligible compared to temperature (A), and it is observed that the compressive load of GF/PP composites increases as the temperature decreases. Additionally, Figure 10c suggests that lower pressure values are desirable for achieving high compressive loads, with the effect of winding speed (C) being minimal compared to pressure (B). The heater temperature remains constant (center point) in Figure 10c.

These observations imply that as the working temperature approaches the upper limit of the defined process window, polymer degradation may occur, leading to a decrease in composite quality and strength. Consequently, operating at the lower temperature limit of the process window is considered safer. Surprisingly, although temperature was expected to have the highest impact on product quality, it is the roller pressure that proves to be most significant in this scenario. This may be attributed to the narrow temperature range of the process window. Since roller pressure directly influences the orientation of fibers within the molten polymer, it is expected to have a significant impact on the strength of the composite. Consequently, operating at the lower roller pressure limits of the process window is preferable for GF/PP composites. Lastly, winding speed does not exert a significant influence on product quality when compared to temperature and pressure.

In summary, operating at higher temperature and lower pressure settings within the process window of GF/PPS leads to higher composite quality. Conversely, lower temperatures and lower pressure settings are preferable for producing high-strength GF/PP composites.

### 3.3. Mechanical, Thermal, and Physicochemical Characterization of the Optimized Filament-Wound Composites

Following the completion of the optimization studies and the production of GF/PPS and GF/PP composites under optimal process conditions, material characterization tests were performed on the optimized filament-wound composites through density measurements, void content analysis, crystallinity measurements, and compression tests. Three cylinders were produced for each composite type, and three test samples were taken from each cylinder, as shown in Figure 7. The results were reported as the average of the measurements from three different sections of each cylinder. Notably, compression tests were not replicated as the optimal point coincided with the 11th trial of the experimental design, yielding compressive loads of 3354.09 N for GF/PPS and 2952.03 N for GF/PP (Table 6). Furthermore, Table 10 presents the crystallinity, density, and void content of all composites along with their corresponding standard deviations.

Density, the mass per unit volume, reflects the distribution of both fiber and matrix in a composite. For thermoplastic composites, density is a key indicator of consolidation quality. Higher density generally signals better fiber impregnation and lower void content, which are crucial for achieving the desired mechanical strength and durability in structural applications. To ensure consistent and near-theoretical density, the precise control of roller pressure, temperature, and winding speed during the filament winding process is crucial. Adequate compaction helps eliminate voids and ensure good fiber impregnation but excessive pressure could lead to fiber damage or distortion. Finding the optimal pressure, maintaining a stable temperature, and holding the winding speed constant to promote even matrix flow will enhance composite quality. During the process, the roller pressure and winding speed were precisely controlled and kept constant. However, even though the heater set temperature was maintained constant, convective heat transfer occurs between the heater tip and the nip point, where the impregnation takes place. As a result, temperature remains the only parameter whose exact value during the process cannot be fully determined.

The theoretical densities presented in Table 1 show that the GF/PP slit tape has a density of 1.656 g/cm^3^, while the GF/PPS slit tape has a higher density of 1.885 g/cm^3^. In contrast, the filament-wound composite densities shown in Table 10 reveal that the density of GF/PP composites has a range of 1.563 to 1.569 g/cm^3^, whereas the GF/PPS composites demonstrate a density range of 1.832 to 1.837 g/cm^3^. The deviation from the theoretical density indicates the presence of voids or incomplete impregnation of the matrix around the fibers during the winding process. GF/PPS composites achieve a density much closer to their theoretical value, indicating more effective consolidation and fiber–matrix bonding during the filament winding process.

Void content represents the trapped air within a composite, which negatively impacts the mechanical properties by introducing weak points that can initiate cracks under stress. Voids reduce the strength, stiffness, and fatigue resistance of composites, making their minimization essential for high-performance applications. The void content results of the GF/PP composites are between 5.2 and 5.6%, as indicated in Table 10. In comparison, the GF/PPS composites exhibit a significantly lower void content of 2.5% to 2.8%. This notable difference implies that the GF/PPS composites experience better consolidation during the filament winding process, leading to reduced void formation. The lower void content in GF/PPS contributes to improved mechanical properties, making it more suitable for high-performance applications.

Reducing void content requires optimization of the heating temperature, roller pressure, and winding speed. A higher temperature promotes matrix flow and fiber wetting, which helps eliminate air pockets. However, excessive temperature can degrade the polymer matrix, particularly in GF/PP, where operating at the upper end of the process window may lead to polymer degradation. Balancing temperature with appropriate pressure ensures void minimization without damaging the fiber structure. Slower winding speeds may also allow for better consolidation, giving more time for the matrix to fill in gaps between fibers.

Crystallinity refers to the degree of molecular order within the polymer matrix. In fiber-reinforced composites, higher crystallinity can enhance stiffness, thermal stability, and chemical resistance. However, excessive crystallinity may lead to increased brittleness, reducing impact resistance, which is vital for dynamic or load-bearing applications. Processing temperature and cooling rates are key factors affecting crystallinity. By carefully controlling the temperature during the winding process and ensuring a gradual cooling phase, the crystallization process can be optimized without compromising the toughness or flexibility of the composite. For GF/PPS, operating at higher temperatures closer to the upper limit of the process window improves crystallinity but cooling must be controlled to avoid excessive brittleness. For GF/PP, lower temperatures may help prevent polymer degradation, preserving the composite’s strength and flexibility. The GF/PP composites show a degree of crystallinity ranging from 57.70% to 59.71%, whereas the GF/PPS composites have a lower range of 48.19% to 53.29% (Table 10). The higher crystallinity in GF/PP suggests enhanced rigidity and thermal stability, but it may also lead to increased brittleness. On the other hand, the lower crystallinity of GF/PPS may be attributed to the crosslinking reaction of PPS. When exposed to temperatures above its melting point in an air environment, crosslinking occurs, which reduces both crystallizability and crystallinity. The rates of change in the degree of crystallinity and crystallizability have been shown to depend on time, temperature, and atmosphere. It has been reported that the curing or crosslinking of PPS leads to a decrease in both crystallinity and crystallizability [45].

This study enhances the understanding of how density, void content, and crystallinity interact with the mechanical properties of filament-wound thermoplastic composites. The interplay between these factors highlights the importance of careful process optimization. Fine-tuning the temperature, roller pressure, and winding speed not only ensures high mechanical performance but also enables the production of composites with tailored properties for specific applications.

## 4. Conclusions

In this study, E-glass fiber-reinforced polypropylene (GF/PP) and polyphenylene sulfide (GF/PPS) cylindrical composites were successfully produced using the filament winding method. The research focused on optimizing key process parameters, including heater temperature, roller pressure, and winding speed, and evaluating their effects on the compressive load performance. The main findings of the study are summarized as follows:

- Optimization of process parameters: Using the Box–Behnken experimental design and response surface methodology (RSM), mathematical models were developed to predict and optimize the process parameters. The optimum processing conditions for GF/PPS and GF/PP composites were identified as a heater temperature of 575 and 275 °C, a roller pressure of 4 bar (321.70 N compaction force), and a winding speed of 1600 and 1200 mm/min, resulting in a compressive load of 3356.99 and 2946.04 N, respectively.
- Time and cost efficiency: The filament winding method demonstrated significant time and cost advantages compared to traditional autoclave processes. Production time was reduced from 3 to 4 h to approximately 30 min, including preliminary preparations. These findings highlight filament winding as a more efficient and cost-effective alternative for manufacturing thermoplastic composites, with potential for broader industrial applications.
- Material-specific observations: The results of this study are specific to the glass fiber-reinforcement used. Properties of different fiber types, as well as their interaction with the polymer matrix, may influence the process and mechanical performance. Additionally, the study explored a temperature range suitable for polypropylene (PP) and polyphenylene sulfide (PPS). For polymers with melting points higher than PPS, further research is necessary to evaluate their compatibility with the filament winding process and to optimize process parameters. Future studies could also explore the use of alternative fiber types and additional process variables such as heating and cooling rates to further enhance performance and broaden the applicability of this technique.
- Industrial scalability: Verifying the reproducibility of the results on an industrial scale is essential to enhance the practical relevance and applicability of the filament winding process in real-world scenarios. However, it is important to note that the choice of heat source plays a critical role in the process. Changes in the heat source may require adjustments to these parameters to ensure optimal performance, highlighting the need for further studies to validate the process under different heating systems.

As a consequence, this research provides a foundational framework for optimizing thermoplastic filament winding processes. By addressing key limitations and demonstrating the efficiency and versatility of the method applied, it establishes filament winding as a viable alternative to traditional methods for producing lightweight, high-strength composite materials suitable for various industrial applications.

## Figures and Tables

**Table 1:** The properties of GF/PP and GF/PPS tapes.

Property	GF/PP	GF/PPS
Density (g/cm^3^)	1.656	1.885
Fiber Content (% by wt.)	70	60
Fiber Volume (%)	45	44
Tape Thickness (mm)	0.25	0.25
Tape Areal Weight (g/m^2^)	435	620
Fiber Areal Weight (g/m^2^)	305	372
Melt Temperature (°C)	173	282
Glass Transition Temperature (°C)	−10	90

**Table 2:** The compaction force corresponding to each roller pressure.

Roller Pressure (bar)	Compaction Force (N)
4	321.70
5	402.12
6	482.55

**Table 3:** Box–Behnken experimental design model [36].

No	A	B	C
1	−1	−1	0
2	1	−1	0
3	−1	1	0
4	1	1	0
5	−1	0	−1
6	1	0	−1
7	−1	0	1
8	1	0	1
9	0	−1	−1
10	0	1	−1
11	0	−1	1
12	0	1	1
13	0	0	0
14	0	0	0
15	0	0	0

**Table 4:** Process factors and levels of the experimental designs for GF/PPS composites.

Factor	Code	−1	0	1
Temperature (T), °C	A	550	575	600
Pressure (P), bar	B	4	5	6
Speed (v), mm/min	C	800	1200	1600

**Table 5:** Process factors and levels of the experimental designs for GF/PP composites.

Factor	Code	−1	0	1
Temperature (T), °C	A	250	275	300
Pressure (P), bar	B	4	5	6
Speed (v), mm/min	C	400	800	1200

**Table 6:** Compression test results of GF/PPS * and GF/PP ** composites (*n* = 3).

No	T_1_ (°C)	P_1_ (bar)	V_1_ (mm/min)	R_1_ (N)	T_2_ (°C)	P_2_ (bar)	V_2_ (mm/min)	R_2_ (N)
1	550	4	1200	3024.17 (±0.05%)	250	4	800	2853.96 (±4.99%)
2	600	4	1200	3058.65 (±2.33%)	300	4	800	2793.13 (±0.66%)
3	550	6	1200	2639.03 (±3.57%)	250	6	800	2761.94 (±1.58%)
4	600	6	1200	3104.77 (±1.18%)	300	6	800	2556.25 (±3.22%)
5	550	5	800	2911.51 (±3.93%)	250	5	400	2623.32 (±3.70%)
6	600	5	800	3160.16 (±0.34%)	300	5	400	2648.16 (±2.62%)
7	550	5	1600	2847.97 (±1.36%)	250	5	1200	2771.56 (±0.33%)
8	600	5	1600	3251.06 (±1.51%)	300	5	1200	2526.77 (±3.91%)
9	575	4	800	3282.81 (±1.85%)	275	4	400	2666.16 (±6.97%)
10	575	6	800	3193.57 (±1.63%)	275	6	400	2769.03 (±4.18%)
11	575	4	1600	3354.09 (±4.83%)	275	4	1200	2952.03 (±1.58%)
12	575	6	1600	3116.96 (±2.17%)	275	6	1200	2493.89 (±2.69%)
13	575	5	1200	3066.75 (±2.56%)	275	5	800	2529.20 (±4.56%)
14	575	5	1200	3051.59 (±3.13%)	275	5	800	2536.90 (±6.75%)
15	575	5	1200	3066.20 (±0.13%)	275	5	800	2548.18 (±5.00%)

* Index 1 for GF/PPS. ** Index 2 for GF/PP.

**Table 7:** Fit statistics for GF/PPS and GF/PP composites.

Specimen	Std. Dev.	Mean	C.V. %	R^2^	Adjusted R^2^	Predicted R^2^	Adequate Precision
GF/PPS	25.18	3075.29	0.8189	0.9929	0.9802	0.8913	35.8940
GF/PP	10.7	2668.7	0.4011	0.9979	0.9942	0.9759	51.6648

**Table 8:** ANOVA results of GF/PPS experimental design.

Source	Sum of Squares	df	Mean Square	F-Value	*p*-Value
Model	4.45 × 10^5^	9	49,416.04	77.91	<0.0001
A	1.66 × 10^5^	1	1.66 × 10^5^	261.54	<0.0001
B	55,342.91	1	55,342.91	87.26	0.0002
C	60.62	1	60.62	0.0956	0.7697
AB	46,494.91	1	46,494.91	73.31	0.0004
AC	5963.36	1	5963.36	9.40	0.0279
BC	5467.76	1	5467.76	8.62	0.0324
A^2^	82,546.63	1	82,546.63	130.15	<0.0001
B^2^	7365.61	1	7365.61	11.61	0.0191
C^2^	63,055.05	1	63,055.05	99.42	0.0002
Residual	3171.23	5	634.25		
Lack of Fit	3023.45	3	1007.82	13.64	0.0691
Pure Error	147.77	2	73.89		
Cor Total	4.48 × 10^5^	14			

**Table 9:** ANOVA results of GF/PP experimental design.

Source	Sum of Squares	df	Mean Square	F-Value	*p*-Value
Model	2.76 × 10^5^	9	30,670.51	267.68	<0.0001
A	29,579.80	1	29,579.80	258.16	<0.0001
B	58,510.55	1	58,510.55	510.66	<0.0001
C	176.51	1	176.51	1.54	0.2696
AB	5245.40	1	5245.40	45.78	0.0011
AC	18,175.35	1	18,175.35	158.63	<0.0001
BC	78,682.04	1	78,682.04	686.71	<0.0001
A^2^	14,516.54	1	14,516.54	126.69	<0.0001
B^2^	72,912.72	1	72,912.72	636.35	<0.0001
C^2^	6407.61	1	6407.61	55.92	0.0007
Residual	572.89	5	114.58		
Lack of Fit	390.56	3	130.19	1.43	0.4371
Pure Error	182.33	2	91.17		
Cor Total	2.77 × 10^5^	14			

**Table 10:** Density, void content, and crystallinity degree of the composites produced at the optimum conditions by filament winding.

Specimen	ρ_c_ (g/cm^3^)	V_C_ %	Xc %
GFPPS-1	1.832 ± 0.012	2.8 ± 0.1	50.73 ± 6.26
GFPPS-2	1.837 ± 0.028	2.5 ± 0.3	53.29 ± 5.95
GFPPS-3	1.833 ± 0.021	2.7 ± 0.2	48.19 ± 5.71
GFPP-1	1.569 ± 0.005	5.2 ± 0.3	59.71 ± 4.01
GFPP-2	1.563 ± 0.039	5.6 ± 0.5	57.70 ± 6.39
GFPP-3	1.566 ± 0.009	5.4 ± 0.5	58.97 ± 7.61

## Data Availability

Data are contained within the article.
